# ADVANCING DEMENTIA RESEARCH THROUGH AN EARLY CAREER SUMMER INSTITUTE

**DOI:** 10.1093/geroni/igae098.1119

**Published:** 2024-12-31

**Authors:** Sam Fazio

**Affiliations:** Chair

## Abstract

In 2020, the National Institutes on Aging (NIA) funded the five-year Alzheimer’s Association Interdisciplinary Summer Research Institute (AA-ISRI) focused on early career researchers interested in psychosocial and public health research related to dementia. This program is designed to equip researchers with the knowledge and tools to advance the field of dementia research. Specifically, the Institute aims to promote skill building and substantive knowledge of emerging leaders, better preparing them to develop the evidence to ultimately improve care and outcomes for persons living with dementia. During each of the first three years of the project, feedback from faculty and trainees underscored areas in which training is more and less beneficial and the importance of facilitating new and ongoing networks. During this symposium, we will 1) describe the inception and development of AA-ISRI; 2) provide insights from a psychosocial track alum on training and the impact AA-ISRI has had on their research and career; 3) provide insights from a public health track alum on training and the impact AA-ISRI has had on their research and career; and 4) detail lessons learned and steps that other organizations can take to develop similar programs, as well as suggestions for faculty working with individual mentees.

